# A35 FORECASTING THE INCIDENCE AND PREVALENCE OF INFLAMMATORY BOWEL DISEASE: A CANADIAN NATION-WIDE ANALYSIS

**DOI:** 10.1093/jcag/gwac036.035

**Published:** 2023-03-07

**Authors:** S Coward, E I Benchimol, C Bernstein, J A Avina-Zubieta, A Bitton, L Hracs, J Jones, E Kuenzig, L Lu, S K Murthy, Z Nugent, A R Otley, R Panaccione, J -N Pena-Sanchez, H Singh, L E Targownik, J W Windsor, G Kaplan

**Affiliations:** 1 University of Calgary, Calgary; 2 The Hospital for Sick Children, Toronto; 3 University of Manitoba, Winnipeg; 4 University of British Columbia, Vancouver; 5 McGill University, Montreal; 6 Dalhousie University, Halifax; 7 Arthritis Research Canada, Winnipeg; 8 The Ottawa Hospital, Ottawa; 9 University of Saskatchewan, Saskatoon; 10 University of Toronto, Toronto, Canada

## Abstract

**Background:**

Canada is currently in the third epidemiological stage in the evolution of IBD: compounding prevalence. A high incidence of IBD, in conjunction with low mortality, leads to a steadily rising prevalence over time. By understanding historical epidemiological trends, we can forecast incidence and prevalence into the future to inform healthcare systems in Canada of the rising burden of IBD to society.

**Purpose:**

To analyze past epidemiological trends in order to forecast the overall incidence and prevalence of IBD, Crohn’s disease (CD), and ulcerative colitis (UC) and stratified by age (<18, 18-64, 65+).

**Method:**

Canadian population-based administrative data was acquired from: AB, BC, SK, MB, QC, and ON. Data were age and sex standardized to the matching year and provincial data aggregated into a representative sample of the Canadian population for prevalence (2002-2014) and incidence (2007-2014: 5-year washout period). Incidence and prevalence (per 100,000 persons) were calculated, with 95% confidence intervals (CI), using Canadian population estimates from Statistics Canada for IBD, CD, UC (IBD-unclassifiable+UC). Autoregressive Integrated Moving Average models were created, and rates forecasted from 2014 to 2035 with 95% prediction intervals (PI). Poisson (or negative binomial) for incidence and log binomial regression for prevalence estimated the Average Annual Percentage Change (AAPC), with 95% CIs, of the forecasted data.

**Result(s):**

The 2014 incidence of IBD in Canada was 28.4 per 100,000 (95%CI: 27.8, 29.0) and forecasted to significantly increase (AAPC: 0.58%; 95%CI: 0.04, 1.04) from 30.0 per 100,000 in 2023 to 32.1 (95%PI: 27.9, 36.3) in 2035. Pediatric onset IBD was 13.9 per 100,000 (95%CI: 13.0, 14.9) in 2014 and is forecasted to significantly increase to 18.0 per 100,000 (95%PI: 15.7, 20.2) in 2035 with an AAPC of 1.23% (95%CI: 0.76, 1.63). Adult and elderly onset incidence rates were forecasted to remain stable. Prevalence of IBD increased between 2002 (389 per 100,000) and 2014 (636 per 100,000) and is forecasted to continue to climb by an AAPC of 2.44% (95%CI: 2.34, 2.53). In 2023, the prevalence of IBD is 825 per 100,000. By 2035 prevalence is forecasted to climb to 1075 per 100,000 (95%PI: 1047, 1103) with 470,000 Canadians living with IBD. Prevalence across all age strata were forecasted to significantly increase. The highest AAPC was seen in the elderly (2.76%; 95%CI: 2.73, 2.79) with a prevalence of 841 per 100,000 (95%CI: 834, 849) in 2014 and forecasted to climb to 1534 per 100,000 (95%PI: 1519, 1550) in 2035.

**Image:**

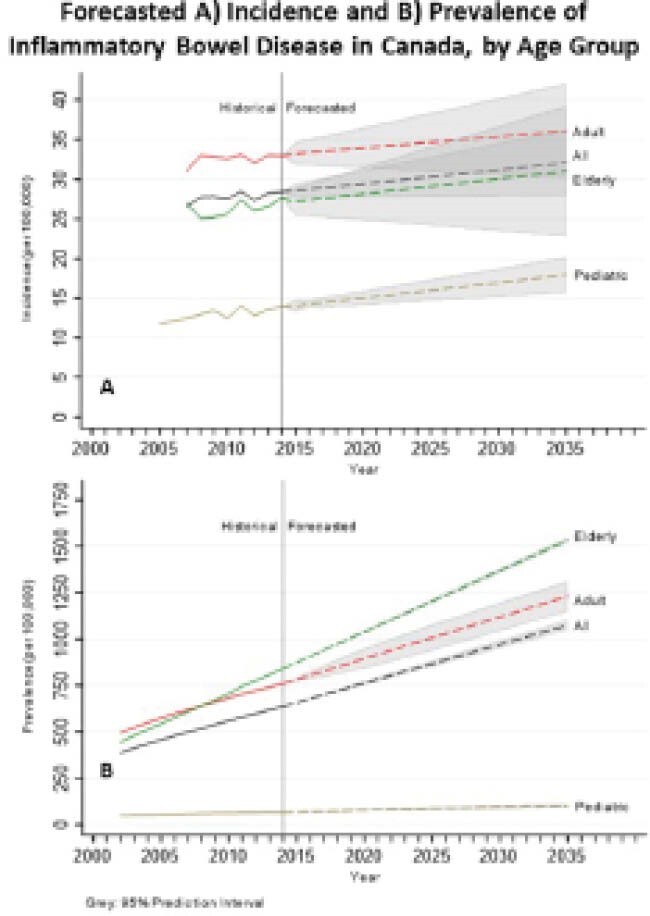

**Conclusion(s):**

Incidence of IBD continues to rise in Canada, driven by pediatric-onset IBD. In 2023, over 320,000 Canadians (0.83%) will be living with IBD. By 2035 prevalence will exceed 1% of the population with approximately 470,000 individuals in Canada with IBD. Future research should establish the environmental determinates of IBD that may influence temporal trends in the incidence of IBD, while healthcare systems adapt to the compounding prevalence of IBD.

**Please acknowledge all funding agencies by checking the applicable boxes below:**

CIHR, Other

**Please indicate your source of funding;:**

The Leona M. and Harry B. Helmsley Charitable Trust

**Disclosure of Interest:**

S. Coward: None Declared, E. Benchimol Consultant of: Hoffman La-Roche Limited and Peabody & Arnold LLP for matters unrelated to medications used to treat inflammatory bowel disease and McKesson Canada and the Dairy Farmers of Ontario for matters unrelated to medications used to treat inflammatory bowel disease., C. Bernstein Grant / Research support from: Unrestricted educational grants from Abbvie Canada, Janssen Canada, Pfizer Canada, Bristol Myers Squibb Canada, and Takeda Canada. Has received research grants from Abbvie Canada, Amgen Canada, Pfizer Canada, and Sandoz Canada and contract grants from Janssen, Abbvie and Pfizer, Consultant of: Abbvie Canada, Amgen Canada, Bristol Myers Squibb Canada, JAMP Pharmaceuticals, Janssen Canada, Pfizer Canada, Sandoz Canada, and Takeda., Speakers bureau of: Abbvie Canada, Janssen Canada, Pfizer Canada and Takeda Canada, J. A. Avina-Zubieta: None Declared, A. Bitton: None Declared, L. Hracs: None Declared, J. Jones Consultant of: Janssen, Abbvie, Pfizer, Takeda, Speakers bureau of: Janssen, Abbvie, Pfizer, Takeda, E. Kuenzig: None Declared, L. Lu: None Declared, S. Murthy: None Declared, Z. Nugent: None Declared, A. Otley Grant / Research support from: Unrestricted educational grants from AbbVie Canada and Janssen Canada, Consultant of: Advisory boards of AbbVie Canada, Janssen Canada and Nestle, R. Panaccione Consultant of: Abbott, AbbVie, Alimentiv (formerly Robarts), Amgen, Arena Pharmaceuticals, AstraZeneca, Biogen, Boehringer Ingelheim, Bristol-Myers Squibb, Celgene, Celltrion, Cosmos Pharmaceuticals, Eisai, Elan, Eli Lilly, Ferring, Galapagos, Fresenius Kabi, Genentech, Gilead Sciences, Glaxo-Smith Kline, JAMP Bio, Janssen, Merck, Mylan, Novartis, Oppilan Pharma, Organon, Pandion Pharma, Pendopharm, Pfizer, Progenity, Protagonist Therapeutics, Roche, Sandoz, Satisfai Health, Shire, Sublimity Therapeutics, Takeda Pharmaceuticals, Theravance Biopharma, Trellus, Viatris, UCB. Advisory Boards for: AbbVie, Alimentiv (formerly Robarts), Amgen, Arena Pharmaceuticals, AstraZeneca, Biogen, Boehringer Ingelheim, Bristol-Myers Squibb, Celgene, Eli Lilly, Ferring, Fresenius Kabi, Genentech, Gilead Sciences, Glaxo-Smith Kline, JAMP Bio, Janssen, Merck, Mylan, Novartis, Oppilan Pharma, Organon, Pandion Pharma, Pfizer, Progenity, Protagonist Therapeutics, Roche, Sandoz Shire, Sublimity Therapeutics, Takeda Pharmaceuticals, Speakers bureau of: AbbVie, Amgen, Arena Pharmaceuticals, Bristol-Myers Squibb, Celgene, Eli Lilly, Ferring, Fresenius Kabi, Gilead Sciences, Janssen, Merck, Organon, Pfizer, Roche, Sandoz, Shire, Takeda Pharmaceuticals, J.-N. Pena-Sanchez: None Declared, H. Singh Consultant of: Pendopharm, Amgen Canada, Bristol Myers Squibb Canada, Roche Canada, Sandoz Canada, Takeda Canada, and Guardant Health, Inc., L. Targownik Grant / Research support from: Investigator initiated funding from Janssen Canada, Consultant of: [Advisory board] AbbVie Canada, Takeda Canada, Merck Canada, Pfizer Canada, Janssen Canada, Roche Canada, and Sandoz Canada, J. Windsor: None Declared, G. Kaplan Grant / Research support from: Ferring, Janssen, AbbVie, GlaxoSmith Kline, Merck, and Shire, Consultant of: Gilead, Speakers bureau of: AbbVie, Janssen, Pfizer, Amgen, and Takeda

